# Cardiovascular damage and comorbidities related to long COVID: pathomechanisms, prevention, and therapy

**DOI:** 10.3389/fcvm.2025.1671951

**Published:** 2025-12-03

**Authors:** Miroslava Kvandova, Peter Balis, Barbora Kalocayova, Jana Vlkovicova, Silvia Dobrodenkova, Angelika Puzserova

**Affiliations:** 1 Institute of Normal and Pathological Physiology, Centre of Experimental Medicine, Slovak Academy of Sciences, Bratislava, Slovakia; 2 Institute for Heart Research, Centre of Experimental Medicine, Slovak Academy of Sciences, Bratislava, Slovakia; 3 Department of Pharmacology and Toxicology, Faculty of Pharmacy, Comenius University in Bratislava, Bratislava, Slovakia; 4 Travel Health Clinic, Bratislava, Slovakia

**Keywords:** COVID-19, endothelium, endothelial dysfunction, flavonoids, micronutrients, post-COVID, epicatechin, vitamins

## Abstract

Long COVID (LC) is a severe acute respiratory syndrome coronavirus 2 (SARS-CoV-2) infection-associated chronic condition and is present for at least 3 months as a continuous, relapsing and remitting, or progressive disease state that affects one or more organ systems, including cardiovascular. Extensive literature supports an association between SARS-CoV-2 infection and cardiovascular complications and increased cardiovascular risk after infection. The cardiovascular sequelae after SARS-CoV-2 infection have not yet been comprehensively characterized. A growing body of evidence suggests that endothelial dysfunction is a central mechanism in COVID-19 and has also been identified as a key pathogenic mechanism in LC. Although considerable progress has been made in characterizing the epidemiology, clinical course, and biology of LC, many questions remain unanswered. The incomplete understanding of the pathomechanisms of LC has hampered the development of targeted therapies to date. Further research and data are needed to develop effective therapeutic and preventive tools. Based on current literature this review aims to provide an up-to-date overview of the pathomechanisms affecting the cardiovascular system and the potential role of selected micronutrients, vitamins and minerals, and flavonoids as preventive and therapeutic strategies in LC.

## 1 Introduction

Coronavirus disease 2019 (COVID-19), first reported in December 2019 in China, is a life-threatening viral infectious disease caused by severe acute respiratory syndrome coronavirus 2 (SARS-CoV-2). Over the past years, the pandemic has resulted in significant morbidity and mortality worldwide, killing more than 7 million people and bringing new challenges, especially regarding the poorly understood long COVID (LC) also called post-acute sequelae of COVID (PASC) or post-COVID-19 syndrome (PCS). LC is the constellation of post-acute and long-term health effects caused by SARS-CoV-2 infection. The overall prevalence of LC in the general population is estimated to be 6%–7% in adults and around 1% in children, and it represents a major public health crisis, it strains health systems and national economies. LC is a single or multiorgan disease state affecting the respiratory, cardiovascular (CV), musculoskeletal, gastrointestinal, immune, and nervous systems, among others. It can be present for weeks to years after the onset of symptoms (1–4). There are currently no validated effective treatment strategies available for LC patients (5). Many millions of SARS-CoV-2 infection survivors, including adults and children, live with this chronic, and often disabling conditions. In recent years, our knowledge of LC has expanded, but it is still largely unknown. For years, there was no universally accepted definition of LC, because of the novelty and diverse expression of this condition (2, 4, 6). According to the 2024 National Academies of Sciences, Engineering, and Medicine (NASEM) definition LC is an infection-associated chronic condition that occurs after SARS-CoV-2 infection and is present for at least 3 months as a continuous, relapsing and remitting, or progressive disease state that affects one or more organ systems (4). The definition situates LC among the larger class of infection-associated chronic conditions. LC manifests in multiple ways and any organ system can be involved. It can follow asymptomatic, mild, or severe SARS-CoV-2 infection. Previous infections may or may not have been recognized. It can be continuous from the time of acute SARS-CoV-2 infection or have a delayed onset for weeks or months after what had appeared to be full recovery from acute infection. It can affect people, regardless of health, disability, socioeconomic status, age, sex, sexual orientation, race, ethnic group, or geographic location. Symptoms can range from mild to severe and can resolve over months or can persist for months or years and many LC patients have had symptoms since early 2020. Yet, no biomarker is currently available that conclusively determines the presence of this condition and diagnosis is a clinical judgment, not a diagnosis of exclusion such other diagnosable conditions can be part of the picture of LC. However, not everyone is at equal risk (risk factors: female sex, repeated infection, more severe infection), LC can affect anyone (4, 7, 8). In the large population-based study of Asadi-Pooya, Akbari (9) LC has significant associations with sex (female), respiratory symptoms at the onset, and the severity of the acute illness (9). Reinfection can trigger *de novo* LC or exacerbate its severity (10, 11).

It is well known that COVID-19 not only affects the respiratory system but results in systemic disease. Cardiovascular damage due to COVID-19 infection may increase mortality, morbidity, and disability thus decreasing the quality of life. Adverse CV manifestations exist in 8%–25% of the overall COVID-19-infected population and this percentage is even higher among intensive care unit (ICU) and non-survival patients (12). On one side, patients with preexisting CV disease and risk factors appear more prone to developing a more severe acute COVID-19 infection and therefore present a high-risk group. On the other side, new-onset CV damage is a common manifestation in patients who tested positive for COVID-19, often lingering for long periods and portends a worse prognosis (12, 13). Although COVID-19 has been initially associated with the respiratory system, CV manifestations have also been reported in COVID-19 patients without respiratory symptoms (14). Even though the primary cause of death of COVID-19 patients is respiratory failure, CV complications are rapidly contributing. Based on that, we will discuss in detail CV manifestations, pathomechanisms responsible for CV damage development, and the possibility of their treatment and prevention based on already published studies and information we know so far.

## 2 Clinical manifestation

The incidence of LC is estimated at 10%–30% of non-hospitalized cases, 50%–70% of hospitalized cases, and 10%–12% of vaccinated cases (5). More than 65 million people worldwide are estimated to have been living with LC since the onset of the pandemic, but the number is still rising. More than 200 symptoms across multiple organ systems have been associated with LC, with the most commonly reported clinical symptoms being post-exertional malaise, fatigue, difficulty concentrating, memory changes, headaches, palpitations, chest pain, dizziness when standing up (lightheadedness), sleep disturbances, difficulty breathing or shortness of breath (dyspnea), cough, problems with taste and smell, bloating, constipation, diarrhea, stomach pain, fever, muscle and joint pains, “pins-and-needles” feelings, rash, and changes in menstrual cycles (Table 1) (4, 15, 16).

**Table 1:** Selected clinical manifestations of long COVID [according to (17)].

Organ system	Symptoms
General	Fatigue
	Fever
	Pain
	Post-exertional malaise
Respiratory	Dyspnea, Exertional dyspnea
	Cough
Cardiovascular	Chest pain
	Palpitations
	Syncope
Neuropsychiatric	Anxiety
	Depression
	Sleep disturbances
	Difficulty concentrating
	Memory changes
	“Brain fog”
	“Pins-and-needles” feelings
	Problems with taste and smell
	Dizziness
Gastrointestinal	Abdominal pain
	Diarrhea
	Obstipation
	Vomitus
	Nausea
Skin	Rashes
	Hair loss
Musculoskeletal	Weakness
	Myalgias
	Arthralgias

Symptoms after COVID-19 infection are wide-ranging and often include persistent cardiac symptoms (18). Long COVID involves multiple adverse outcomes, with common new-onset conditions including CV, thrombotic, and cerebrovascular disease, type 2 diabetes mellitus (DM), myalgic encephalomyelitis/chronic fatigue syndrome, and dysautonomia, especially postural orthostatic tachycardia syndrome (POTS) (5). A large prospective study in Wuhan (China) of hospitalized COVID-19 patients showed that up to 68% of survivors reported at least one symptom at 6-month follow-up after the onset of COVID-19 (19). Potential acute CV complications of SARS-CoV-2 infection include myocardial injury, myocarditis/pericarditis, acute coronary syndrome, left and right heart failure, pulmonary hypertension, venous thromboembolism, cerebrovascular disorder, stroke, Takotsubo syndrome, cardiac arrhythmia, sudden cardiac arrest and chronic CV complications involve left heart failure, right heart failure, recurrent myocarditis/pericarditis, acute coronary syndrome (post-infection), hypertension, thromboembolism, cerebrovascular disorder, stroke (post-infection), cardiomyopathy, POTS, arrhythmias, and sudden cardiac arrest (20–22).

There are many theories regarding the cause of LC, however, the causes are likely multifactorial and potentially overlapping, including ongoing viral infection (viral persistence), which contributes to ongoing inflammation and immune response, immune system dysregulation, with or without reactivation of latent viruses (including herpesviruses such as Epstein–Barr virus and human herpesvirus 6), microthrombi (micro-clots), endothelial dysfunction (ED), microbiota dysbiosis, systemic fibrosis, autoimmunity, and autonomic dysfunction (5, 7, 16). Long COVID is not a psychosomatic condition but is an organic post-acute infection syndrome (known also as PAIS) with clear physiological dysfunction that is often not consistently apparent using standard medical diagnostic tests (7). The importance biomarker research that can be used for the diagnosis of LC will not only help establish the diagnosis but will also be helpful for objectively defining treatment responses (5).

COVID-19 patients may present with non-specific symptoms, such as dyspnea and fatigue that may have a cardiac origin (20). Also, palpitations, chest pain, dyspnea, and syncope are the most common symptoms among LC patients (13). The clinical manifestations of CV damage in COVID-19 patients include myocarditis (including fulminant myocarditis) and pericarditis, hypertension, arrhythmias, myocardial injury (evidenced by elevated troponin, creatine kinase, NT-proBNP levels), and heart failure, coronary heart disease, stress cardiomyopathy (Takotsubo cardiomyopathy), stroke, blood coagulation abnormalities (coagulation evidenced by elevated level of D-dimer), and also dyslipidemia (13, 23).

Cardiovascular complications have been described in the acute phase of COVID-19. Yet, clinical studies show that the risk of incident CV disease (CVD) extends well beyond the acute phase of COVID-19. However, the clinical manifestation of LC is very variable, and the range of symptoms is wide and individual, depending on the course of an acute infection (15). Selected reported CV symptoms, including frequency and presence at the time of study observation, are mentioned in Table 2 below. However, there are large variations in estimates of the prevalence and incidence of cardiac symptoms and CVD after recovery from acute SARS-CoV-2 infection because of the differences in study populations, methods, follow-up periods, and sample sizes.

**Table 2:** Frequency and presence of selected cardiovascular symptoms in clinical studies after the onset of COVID-19 illness or discharge from the hospital.

Symptom	Occurrence	Presence	References
Palpitations	9%	6 months	(19)
After physical load	12.6%	1 month	(24)
After physical load	5.2%	3 months	(24)
At rest	4.8%	1 month	(24)
At rest	0.4%	3 months	(24)
	6%	1 month	(25)
	11%	2 months	(25)
	68.8%	1–6 months	(26)
	4.8%	3 months	(27)
	14.4%	9.3–11.0 weeks	(28)
	11%	3–6 months	(9)
	8%	6–12 months	(9)
	36%	3–6 months	(29)
Chest pain/chest discomfort	21.7%	2 months	(15)
	11.9%	1 month	(24)
	4.8%	3 months	(24)
	18%	1 month	(25)
	13.1%	2 months	(25)
	54.7%	1–6 months	(26)
	12.3%/14.1%	3 months	(27)
	19.4%	9.3–11.0 weeks	(28)
	11%	3–6 months	(9)
	9%	6–12 months	(9)
	5%	6 months	(19)
	33%	3–6 months	(29)

## 3 Cardiovascular comorbidities

Based on the available evidence in the scientific literature, it seems that SARS-CoV-2 infection prominently affects the CV system and new onset comorbidities may develop, or existing comorbidities may be worsened by COVID-19. Comorbidities are an important prognostic factor. Infection with SARS-CoV-2 has adverse outcomes for individuals with particular comorbid diseases, e.g., hypertension, obesity, type 2 DM, and other CVDs and this may be due to the presence of pre-existing ED and systemic inflammation in subjects with these conditions (30, 31).

Cardiovascular/cardiometabolic comorbidities such as coronary artery disease (ischemic heart disease, coronary heart disease), hypertension, and DM dramatically increase the risk of in-hospital mortality in COVID-19 patients (32). Age, diabetes, chronic obstructive pulmonary disease, and chronic kidney disease are independent predictors of the severity of COVID-19 (33). Also, the association between hypertension and COVID-19 severity is now well recognized (33). SARS-CoV-2 infection may cause acute CV damage and also may lead to post-recovery sequelae (33).

Thus SARS-CoV-2 infection may cause persistent CV symptoms following recovery from acute COVID-19 (20). Many studies have highlighted several severe symptoms related to CV system damage as a consequence of acute COVID-19 infection, where more clinical manifestations and disabilities were observed than usually observed after viral infection (12). Mild cases of COVID-19, defined as not requiring hospitalization in the acute phase, were originally thought to have no long-term consequences. Now, the CV complications of acute COVID-19 are well described, but the post-acute CV manifestations of COVID-19 have not yet been comprehensively characterized. Non-hospitalized patients in the acute phase of COVID-19 were younger, had lower CVD burden, and recovered at home, have not been studied sufficiently, and should be the target of future research concerning long-term CV damage and complications (33).

In a US study including more than 150 000 individuals, 1 year after SARS-CoV-2 infection, a significantly increased risk of a variety of CVDs, including heart failure, dysrhythmias, myocardial infarction, and stroke, independent of the severity of initial COVID-19 presentation was observed (34). The risks and burdens of CVDs were evident even among those patients whose acute COVID-19 did not necessitate hospitalization (the majority of people with COVID-19) and were also evident in people without any CV disease before exposure to COVID-19 (34).

SARS-CoV-2 infection can cause significant vascular pathology, cardiac injury, and acute and chronic CV complications. COVID-19 is an independent risk factor for CVDs and there is a persistent post-acute risk of adverse CV outcomes, including ischemic stroke and acute myocardial infarction (20).

It was reported that COVID-19 patients with CV manifestations have a worse course of disease with a more frequent need for ICU hospitalization and increased mortality (12). COVID-19 mortality is increased by CV comorbidities and pre-existing risk factors such as hypertension, coronary heart disease, DM, and obesity (23, 33, 35). It was reported that the increased prevalence of cardiac diseases, such as heart failure, atrial fibrillation, and coronary artery disease, in hospitalized COVID-19 patients negatively affects prognosis due to thromboembolic events and septic shock compared to COVID-19 patients without a history of cardiac disease. Approximately 40%–50% of hospitalized patients had some CV or cerebrovascular diseases (33).

A large cohort study provided by Ayoubkhani, Khunti (36) showed that during 140 days, one-third of the already discharged COVID-19 patients were re-admitted, and more than one patient from 10 did not survive. 4.8% of discharged patients were re-admitted to the hospital during eight months due to severe CV events, including myocardial infarction, heart failure, stroke, and arrhythmia (36). Other studies also showed arrhythmias and persistent myocardial injury after acute COVID-19 (33, 34). In 22% of discharged severe COVID-19 patients with troponin elevation myocardial injury or ischemic heart disease was found two months after discharge from the hospital. Moreover, ischemic heart disease was found in COVID-19 patients without a previous history of coronary disease (37). It was shown that myocardial infection increases the risk of CV events and complications during virus infections such as influenza. Mortality of influenza-infected patients with myocardial infarction was significantly increased during hospitalization. Moreover, it was observed that those patients had an increased incidence of CV complications in the post-acute state, leading to a higher rate of 30-day hospitalization (38).

One study of persistent cardiac symptoms, including exertional dyspnea, following recovery from mild COVID-19, showed that despite the absence of elevated troponin, diffuse myocardial edema was seen on cardiac magnetic resonance imaging (18). In this study, after a mean follow-up period of 329 days post-COVID-19 infection, 5% of previously asymptomatic participants reported new cardiac symptoms.

COVID-19 infection is affecting arrhythmias development as well (39). It was found that beyond the first 30 days of COVID-19, an arrhythmia risk occurrence is eight times higher (40). Similarly, it was found that 6.1% of COVID-19 patients reported symptomatic palpitations or persistent tachycardia 28 days after acute COVID-19 infection (41), and 11% complained of intermittent palpitations episodes between 3 and 6 months after illness (9). Arrhythmias, including ventricular, were reported in 27% of discharged patients 3 months after hospitalization for COVID-19 (42). In the diagnostic work-up of long COVID patients presenting with palpitations or ventricular arrhythmias, a standard 12-lead ECG with analysis of QRS morphology and the ventricular arrhythmia axis can aid in differentiating idiopathic ectopy from arrhythmias secondary to myocarditis or structural heart disease. Several electrocardiographic indices have been proposed to predict the site of origin of premature ventricular complexes (43, 44). This approach facilitates risk stratification and guides the need for further imaging evaluation. POTS was reported in 4 of 6 patients in a cohort of patients with orthostatic intolerance associated with COVID-19. Autonomic symptoms were present at COVID-19 onset in 5 of the 6 patients in this series, in the remaining patient began 6 weeks following the onset of typical COVID-19-related symptoms. POTS occurrence can be a result of autonomic dysfunction (dysautonomia) (45). It has been increasingly shown that after recovery from COVID-19, survivors may exhibit persistently elevated blood pressure (BP) and sustained tachycardia, which can manifest in various forms such as sinus tachycardia at rest, inappropriate sinus tachycardia (IST) or postural orthostatic tachycardia syndrome (32, 33, 46).

Heart failure in LC patients was presented four times more than in corresponding controls. Up to 55% of patients develop abnormal echocardiograms during acute COVID-19 infection, and those complications may persist (47), and even can lead to adverse ventricular remodeling in 29% of patients three months after post-infection (48). In a prospective, multicenter observational study from Austria echocardiographic data at 60- and 100-days post COVID-19 infection showed a high prevalence of diastolic dysfunction (49). After an average follow-up of 5 months of 160 patients who had been discharged from the ward or the outpatient clinic after a diagnosis of COVID-19, most still exhibit morpho-functional alterations that involve the right (RV) and left ventricle (LV), identified by right ventricle dilatation, increased pressure in the pulmonary circulation, and bi-ventricular systolic-diastolic dysfunction (50). In the echocardiographic study of 100 dyspneic patients requiring hospitalization due to COVID-19 infection, the most common finding at admission was RV dilatation with or without dysfunction (39%), followed by LV diastolic dysfunction (16%) and LV systolic dysfunction (10%) (51). Subclinical RV dysfunction occurred even in patients without previously identified risk factors for CV disease 30 days after hospital discharge following treatment for moderate to severe COVID-19 pneumonia (52). It was shown that cardiac impairment correlates with LC severity level (53). In COVID-19, increased cases of Takotsubo cardiomyopathy were also observed (54). After this syndrome, patients can develop a persistent, long-term heart failure phenotype with a significant impact on quality of life (55).

Myocarditis is developed during the acute phase of COVID-19 infection, but existing evidence suggests that it can persist. In 60% of patients with already existing inflammation still lasting myocarditis ten weeks after the initial COVID-19 infection and was independent of previous comorbidities (56). The risk of myocarditis occurrence is present in highly trained athletes as well. In a study involving 26 competitive athletes who had mild or asymptomatic COVID-19 infection 15% had CMR findings suggestive of myocarditis after recommended quarantine (11–53 days) (57). It is important to note that differential diagnoses must be considered in patients presenting with chest pain, dyspnea, or elevated troponins after SARS-CoV-2 infection. Myocarditis can mimic acute coronary syndrome, pericarditis, and specific cardiomyopathies, therefore, cardiac magnetic resonance, coronary imaging, and biomarker assessment remain key to distinguishing these entities (58). For patients with acute or subacute myocarditis presenting with reduced ejection fraction or sustained arrhythmias, temporary protection using a wearable cardioverter-defibrillator (WCD) may be considered until recovery. This approach offers arrhythmic protection during the vulnerable inflammatory phase (59).

A new onset of arterial hypertension after COVID-19 recovery was observed as well. Even one month after acute infection both systolic and diastolic BP were significantly increased compared to the values at admission to the hospital (60). 21.6% of hospitalized patients had uncontrolled BP shortly after hospital discharge (approximately 3–4 weeks) requiring therapeutic change (61). By injuring kidney epithelial cells, SARS-CoV-2 also impairs BP regulation (62). Long COVID is one of the factors influencing CV risk in patients with hypertension (63).

Cardiometabolic diseases such as DM can also develop due to COVID-19 infection. Montefusco, Ben Nasr (64) observed glycemic alterations not only in the acute phase of COVID-19 but also long after remission of the COVID-19 disease. They detected glycemic abnormalities in patients, who recovered from COVID-19, for at least two months (64). Similarly, a higher level of C-peptide was found with a simultaneous reduction in the fasting glucose level, suggesting an insulin resistance development at the 3- and 6-month follow-up after discharge (65). New-onset DM was reported in 58 cases from 1733 COVID-19 patients without a previous history of DM 6 months after acute infection (19). Follow-up of youth with presymptomatic type 1 DM demonstrated that the COVID-19 pandemic was associated with an accelerated progression to clinical disease and that this acceleration was confined to those with COVID-19 (66). While comorbidities, such as diabetes, obesity, chronic CVDs, chronic kidney disease, cancer, and organ transplantation, are well-recognized determinants of increased severity and mortality related to acute COVID-19, their association with LC in those who have recovered remains to be determined (67).

**Figure 1:**
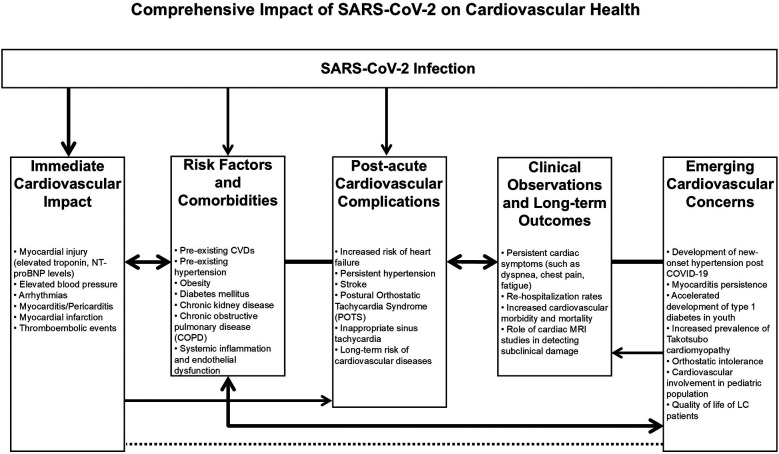
Comprehensive impact of SARS-CoV-2 on cardiovascular health. The SARS-CoV-2 infection may be the primary trigger for all subsequent events and health issues, including direct cardiovascular damage, which leads to increased mortality and morbidity in patients (Immediate Cardiovascular Impact, Risk Factors, and Comorbidities). SARS-CoV-2 infection can worsen pre-existing risk factors and co-morbidities, further escalating the severity of the disease and the risk of developing cardiovascular complications (Risk Factors and Comorbidities, Post-acute Cardiovascular Complications). Cardiovascular damage caused by the infection leads to acute and post-acute complications that can persist long after the acute phase of COVID-19 (Post-acute Cardiovascular Complications, Clinical Observations, and Long-term Outcomes). These complications impact diagnostic outcomes, including cardiac imaging and biomarkers, crucial for monitoring and patient follow-up (Clinical Observations and Long-term Outcomes, Emerging Cardiovascular Concerns). The long-term management of patients who have recovered from COVID-19 and are at risk of cardiovascular complications is essential. Clinical management must be tailored to the individual needs of patients. Emerging cardiovascular concerns necessitate adjustments in management to ensure a comprehensive understanding of COVID-19's impact on the cardiovascular system and patient care (Emerging Cardiovascular Concerns). CVDs, cardiovascular diseases; LC, long COVID; NT-proBNP, N-terminal pro–B-type natriuretic peptide.

Care strategies for those surviving the acute episode of COVID-19 should include attention to CV health and disease (Figure 1) and increased awareness of potential CV causes of non-specific LC symptoms is essential.

## 4 Pathomechanisms from the viewpoint of the cardiovascular system

The mechanism(s) that underlie the association between COVID-19 and the development of CVDs in the post-acute phase of the disease are not entirely clear. Yet, LC research focuses on several pathological mechanisms. Hence, multiple pathomechanisms may be involved in acute COVID-19 CV involvement, including immune-mediated mechanisms, ischemia, hypoxia due to demand-supply mismatch, and thrombosis, among others (39). Cardiovascular damage during acute infection may be because of the virus toxicity or due to a dysregulation of the inflammatory or immunological responses leading to a cytokine storm (33). Several studies have demonstrated the ability of SARS-CoV-2 to cause CV damage, both indirectly and directly. Two important pathomechanisms of CV impairment may thus be the direct virus-mediated cytotoxicity as well as indirect hyperimmune responses of the body to infection. As we mentioned above, several mechanisms participate in LC pathophysiology as well, including viral persistence, immune dysregulation, mitochondrial dysfunction, ED, and microbiome dysbiosis. However, the pathophysiological mechanisms of LC are still being elucidated and it is unlikely that a single mechanism can explain the wide and heterogeneous set of symptoms and diseases involving different organ systems. LC likely represents a disease with many subtypes, each of which may have its risk factors, biological mechanisms, and disease trajectory and may respond differently to treatment. There are likely many factors that shape the human host response during the acute phase of SARS-CoV-2 infection and may trigger the pathomechanisms that ultimately produce LC phenotypes (3).

The persistent risk of CV events and diseases has been supposed to be due to several factors, including persistent virus in the body, myocardial injury, or ongoing immunological and inflammatory effects (20). Yet, LC symptoms were associated with persistent SARS-CoV-2, and viral persistence was detected across multiple tissue samples (68, 69). Persistence of the SARS-CoV-2 virus can trigger a dysregulation of the immune system, followed by increased release of inflammatory cytokines and abnormal endothelial damage, ultimately leading to the development of chronic inflammation, vascular damage, hypercoagulability, microthrombosis, and multiorgan symptoms (68). Therefore, putative mechanisms include lingering damage from direct viral infection of cardiomyocytes and endothelial cells and viral persistence in the CV system (16, 70, 71). SARS-CoV-2 viral proteins and/or RNA have been found across the CV system (2, 5). Stahl, Brasen (70) showed the presence of viral particles directly in vascular endothelium (70). The disruption of the CV system encompasses ED and subsequent down-stream effects, and increased risks of deep vein thrombosis, pulmonary embolism, and bleeding events (5). Several studies have found elevated levels of autoantibodies in LC, including autoantibodies to angiotensin-converting enzyme 2 (ACE2, the receptor for SARS-CoV-2 entry), β2-adrenoceptor, muscarinic M2 receptor, angiotensin II (Ang II) AT_1_ receptor and angiotensin 1-7 MAS receptor (5). Autoantibodies that target the tissue, such as vascular endothelium, extracellular matrix components, coagulation factors, and platelets have been found in some COVID-19 patients (5). LC research has also found mitochondrial abnormalities (5). Cell death of cardiomyocytes and other changes affecting myocardial integrity, such as fibro-fatty replacement, displacement of desmosomal proteins, modulation of the expression of cardiomyocyte ion channel proteins, myocardial fibrosis, were observed (67) and leads to arrhythmic sequelae in acute and post-acute COVID-19 (72). Many studies have also found ED, and coronary and cerebral blood flow abnormalities (5). Eberhardt, Noval (71) showed that in the heart the virus infects coronary vessels, targeting coronary artery atherosclerotic plaque macrophages and inducing plaque inflammation that could trigger acute CV complications and increase the long-term CV risk (71).

Virus infection depends on the entry of the virus into the cell using already existing mechanisms of the host. It was described that possible responsible pathomechanisms leading to CV complication development are triggered by ACE2, which is a key enzymatic component of the renin-angiotensin-aldosterone system (RAAS). The RAAS is an important regulator of CV physiology. ACE2 catalyzes the conversion of Ang II, a peptide with multiple actions that promote CVDs, and generates angiotensin 1-7 (Ang 1-7), which has opposite cellular effects and antagonizes the effects of Ang II (73, 74). On one side, Ang II is a well-described vasoconstrictive agent that enhances the sympathetic tone and activates pro-inflammatory pathways resulting in ED. On the other hand, Ang 1-7/angiotensin type 2 receptor (AT_2_R) has a vasodilatory effect, inhibits inflammation and fibrosis, and plays a cardioprotective role (75). The significance of ACE2 in the CV system is in negative regulation of the RAAS, whose activation is associated with the development of CV comorbidities such as hypertension due to increased Ang II concentration, Ang II-induced vasoconstriction, sodium retention, induction of oxidative stress (OS), and induction of inflammation and fibrosis development (75, 76).

SARS-CoV-2 uses the membrane-bound ACE2 (receptor) as a coreceptor for entry (73). Another receptor used for virus entry into cells is CD209l/L-SIGN. Moreover, virus entry is facilitated by the interaction of CD209l/L-SIGN and ACE2 receptors (77). CD147 is another potential alternative receptor for SARS-CoV-2 entry into cells (78). Before binding to ACE2 receptors, viral spike proteins must be primed by cellular proteases, specifically, transmembrane serine protease 2 (TMPRSS-2). Therefore, COVID-19 appears to affect cells that co-express ACE2 and TMPRSS-2. Both the ACE2 receptor and TMPRSS-2 gene are expressed on endothelial cells and likely explain why COVID-19 infection produces widespread ED (13, 79). The cell-surface ACE2 receptor internalizes on binding to the SARS-CoV2 spike protein, leading to ACE2 receptor down-regulation (80).

In acute SARS-CoV-2 infection, ACE2 is a key factor in cardiomyocyte invasion and systemic inflammation development. Autopsy of the hearts of COVID-19 patients showed decreased expression of ACE2. Without any compensatory action, this reduction can lead to the RAAS tilting towards the harmful axis induced by Ang II (81). Thus, the interaction between the SARS-CoV-2 spike protein and the ACE2 receptor leads to the downregulation of ACE2, resulting in the local enhancement of Ang II concentration and stimulation of the AT_1_R. The vasoconstrictive effect of Ang II is even more pronounced as the vasodilatation of the RAAS axis – ACE2/Ang 1-7/AT_2_R is attenuated, and the expression/activation of endothelial nitric oxide synthase (eNOS) is reduced due to OS. Reactive oxygen species (ROS) production is increased via COVID-19-induced mitochondrial damage and activation of NADPH oxidase (82). The relationship between inflammation and OS was observed in COVID-19 patients. Stimulation of the RAAS can also elicit pro-inflammatory and profibrotic responses and contribute to CV remodeling (83). It is also hypothesized, that tetrahydrobiopterin (BH4) deficiency and OS may be implicated in LC patients (84). However, the impact of RAAS and OS on LC symptoms and CV manifestations needs further evaluation.

SARS-CoV-2 can cause the immune system to strongly release various cytokines and chemokines, which may lead to extensive ED among others (13). SARS-CoV-2 infection triggers inflammation in the microvascular endothelial cells in multiple organs, including the heart (85) as markers of endothelial inflammation - IL-6, TNF-α, IL-1β, IL-15 – were increased in the lungs of COVID-19 non-survivors (86). Significant inflammation with endotheliitis can lead to disseminated intravascular coagulation (DIC), and thrombosis of small and large vessels with tissue necrosis or infarction. The concentrations of IL-6, TNF-α, and IL-1 in patients with LC were substantially elevated for prolonged periods (13).

Endothelial cells have emerged as key players in COVID-19 and LC pathologies. Endothelial cells line the inner layer of blood vessels that keep micro- and macro-vascular health by sensing pathogen/danger signals and secreting vasoactive (e.g., vasorelaxing and vasoconstricting) molecules and are essential for maintaining tissue homeostasis (23, 87). The endothelium regulates vascular tone, circulation of blood cells, vascular permeability, inflammation, and hemostasis by controlling the fine balance between vasodilatory and vasoconstrictory, pro-proliferative and anti-proliferative, pro-thrombotic and anti-thrombotic, pro-oxidant and antioxidant, fibrinolytic and anti-fibrinolytic, and pro-inflammatory and anti-inflammatory responses (23). Physiologically the endothelium maintains an antioxidant, anti-inflammatory, and anti-thrombotic interface (23). Endothelial dysfunction is considered an early sign of atherosclerosis and an early predictor of future CV events (88). According to CV complications, ED can promote chronic inflammation and disease processes like thrombosis and atherosclerosis (82). Several different viral species, such as dengue, Ebola, Marburg, Lassa fever, yellow fever and influenza viruses, cytomegalovirus, and coronaviruses are known to have effects on the endothelium (30). Increasing evidence suggests, that SARS-CoV-2 infection affects the pulmonary and extrapulmonary vasculature, and leads to ED (endotheliitis, endothelialitis, and endotheliopathy), which is thus a unified key mechanism in the pathogenesis of COVID-19 (23, 89). Also, COVID-19 is considered as an endothelial disease. In CV complications, ED plays a fundamental role (23). The LOCHINVAR study also highlights a potential long-term cardiovascular risk in COVID-19 survivors, as evidenced by elevated blood pressure and impaired endothelium-dependent flow-mediated dilation observed 12 months after infection (90). Endothelial dysfunction can thus mediate the adverse influence of SARS-CoV-2 on CV health.

The prothrombotic phenotype and diffuse intravascular coagulation observed in COVID-19 also reflect ED (91). Pretorius, Vlok (92) found amyloid microclots in plasma samples from LC patients that were resistant to fibrinolysis (92). In addition to thrombosis in larger vessels, microthrombi can also play an important role in CV pathology, but the mechanism behind SARS-CoV-2-associated coagulopathy is not well understood (20). Varga, Flammer (85) found direct viral infection of the endothelial cells and diffuse endothelial inflammation, with endothelial and inflammatory cell death in an autopsy of patients with COVID-19. Their findings suggest that SARS-CoV-2 infection facilitates the induction of endotheliitis in several organs as a direct consequence of viral involvement and the host inflammatory response and can explain the systemic impaired microcirculatory function in different vascular beds and their clinical sequelae in COVID-19 patients (85). Stahl, Brasen (70) found abundant and seemingly intact viral particles in the bowel endothelium of a 43-year-old male patient about 8 weeks after initial SARS-CoV-2 infection when the virus was already undetectable in respiratory and blood specimens. In this case study histological examination revealed severe endothelialitis and multiple microthrombi in the venous vascular bed (70). Viral persistence in the penile tissue collected from patients who underwent surgery for penile prosthesis surgery due to severe erectile dysfunction has also been documented, as has an increased risk of erectile dysfunction in men with a history of COVID-19 infection and likely resulting from ED (79). The infection of endothelial cells or pericytes could lead to severe microvascular and macrovascular dysfunction. In association with immune hyperreactivity, it can potentially destabilize atherosclerotic plaques and explain the development of acute coronary syndromes (81). Haffke, Freitag (93) also found ED and altered endothelial biomarkers in patients with LC (93).

Endothelial cell integrity is significantly affected by the cytokine storm induced by COVID-19. This can trigger an endothelial-to-mesenchymal transition (94), where the loss of endothelial-specific markers and gaining of a mesenchymal or myofibroblastic phenotype contribute to fibrosis development due to the generation of activated myofibroblasts (95). Additionally, the endothelial glycocalyx, a cellular microstructure of proteoglycans and glycoproteins on the endothelial cell surface, is disrupted in COVID-19 patients (96–98). This disruption allows for the interaction of the ACE2 receptor with the spike protein of the SARS-CoV-2 virus (99). The intact glycocalyx structure of the endothelium could repel SARS-CoV-2 (23). Moreover, the plasma from COVID-19 patients promotes glycocalyx shedding and redox imbalance in endothelial cells, and low molecular weight heparin treatment potentially inhibits glycocalyx disruption (96). Syndecan-1 (SDC-1), a marker of endothelial glycocalyx degradation was found in COVID-19 patients (23). In convalescent COVID-19 patients, significantly elevated SDC-1 levels were detected after a median of 88 days after symptom onset compared to healthy controls, whereas no difference was found when compared to SDC-1 levels of hospitalized patients undergoing acute COVID-19 disease (100). Therefore, treatments aimed at maintaining endothelial glycocalyx integrity may be promising therapeutic tools for managing COVID-19 (23) and LC. COVID-19-induced “cytokine storm” can disrupt endothelial cell integrity and affect barrier function via modulation of junctional proteins (VE-cadherin, ZO-1, β-catenin, and gap junction proteins), leading to the extravasation of inflammatory and immune cells (101). This is further supported by the study of Yang, Huang (102) in which the significant induction of cytokines, chemokines, and adhesion molecules (such as TNF-α, IL-1β, MCP-1, CXCL1, ICAM-1, VCAM-1) in human brain microvascular endothelial cells by SARS-CoV-2, suggesting an activation of the vascular endothelium in the brain. Moreover, the authors demonstrated that SARS-CoV-2 infection could increase the blood-brain barrier permeability by downregulating as well as remodeling the intercellular tight junction proteins. In this study, SARS-CoV-2 infection up-regulated expression of genes enriched in signaling pathways relevant to inflammatory response (such as NF-κB and NOD-like receptor signaling pathway) (102). In addition, SARS-CoV-2 infection leads to the release of pro-angiogenic factors, such as vascular endothelial growth factor A (VEGF-A) (23). In the lungs of patients with COVID-19, the amount of new vessel growth was 2.7 times as high as that in the lungs of patients with influenza A virus subtype H1N1 (103). COVID-19 infection is also associated with endothelial senescence, in which increased secretion of pro-inflammatory cytokines, pro-coagulatory factors, and VEGF was seen (23). On the other hand, a long-lasting reduction in sublingual vascular density, specifically affecting small capillaries, was found in LC patients compared to healthy controls 18 months after infection (104). Studies have also reported that spike glycoprotein and nucleocapsid protein of SARS-CoV-2 cause endothelial cell activation, evidenced by increased expression of cytokines/chemokines (23, 105). Together, emerging evidence suggests that endothelial activation is an early hallmark of multiorgan damage in patients with COVID-19. It was found that the nucleocapsid protein of SARS-CoV-2 significantly activated human endothelial cells through Toll-like receptor 2/NF-κB and mitogen-activated protein kinase signaling pathways (105). In addition, reduced flow-mediated dilation suggesting ED was observed in hospitalized COVID-19 patients (91). It is reported that COVID-19 patients at admission had a higher number of circulating endothelial cells than COVID-19-negative subjects (106). Moreover, circulating endothelial cells infected with SARS-CoV-2 function as vectors for viral dissemination by forming clusters that migrate into the circulation and reach distant organs. The cell clusters and ED might contribute to the various thromboembolic pathologies observed in COVID-19 by inducing the formation of intravascular microthrombi (107). Endothelial cell depletion from the vascular luminal surface reduces nitric oxide (NO) production, which results in greater contractility and impaired endothelium-dependent vasorelaxation after SARS-CoV-2 infection (107). Nagashima, Mendes (31) showed that pyroptosis an inflammatory form of programmed cell death could contribute to endothelial cell death after SARS-CoV-2 infection. However, future studies are needed to elucidate the exact effects of SARS-CoV-2 on endothelial function (31).

Oxidative stress is an important determinant in the development of ED (88). As mentioned above, increased OS was observed in COVID-19, in which excessive production of mitochondrial ROS, ROS derived from NADPH oxidase activation as well as eNOS uncoupling is possible (23). It is undeniable that in LC pathogenesis, inflammation plays a critical role. In patients with COVID-19, the correlation between IL-6 as a marker of inflammation and OS was observed. Moreover, IL-1β secreted by SARS-CoV-2-infected monocytes partially depended on lipid peroxidation (ROS-mediated oxidative degradation of lipids). Oxidative stress and inflammatory state were still present in recovered patients (108). It was discussed that persistent OS and excessive inflammation are key factors linking the acute phase of COVID-19 and the development of LC (109, 110). Oxidative stress affects all aspects, such as inflammation, ED, neuroinflammation, disruption of neurotransmitter secretion, and coagulopathy development, which are responsible for the induction of persistent LC symptoms. It was suggested that OS could be essential in the causal nexus of immuno-endothelial-neurological damage during LC (110).

Oxidative stress is induced during acute infection and often persists even after the acute infection, which contributes to sympathetic neuronal system dysfunction via alpha-adrenergic signaling modulation. It affects the perfusion of coronary and cerebral arteries and can also contribute to the development of CV comorbidities reported in LC patients (111). Moreover, ED triggered by OS in LC patients was found, and its prevalence was correlated with the severity of acute infection, especially pulmonary dysfunction. Here it was reported that the occurrence of ED depends on the sex. Females can be protected from ED due to their genetic and hormonal composition (112). Still, additional research should be provided to clarify these contexts. Also, age can play a significant role here as well. Considering OS as one of the critical factors in LC development, antioxidant defense failure as a consequence of physiological aging may explain the increased occurrence of LC in the elderly and patients with already pre-existing cardiometabolic diseases, where is known that OS and inadequate antioxidant response due to decreased expression and/or activation of antioxidant enzymes are present (113). Thus, enhanced ROS production after SARS-CoV-2 infection will elicit long-term deleterious effects on endothelial cells, including decreased NO bioavailability and flow-mediated vasodilation (23).

**Figure 2:**
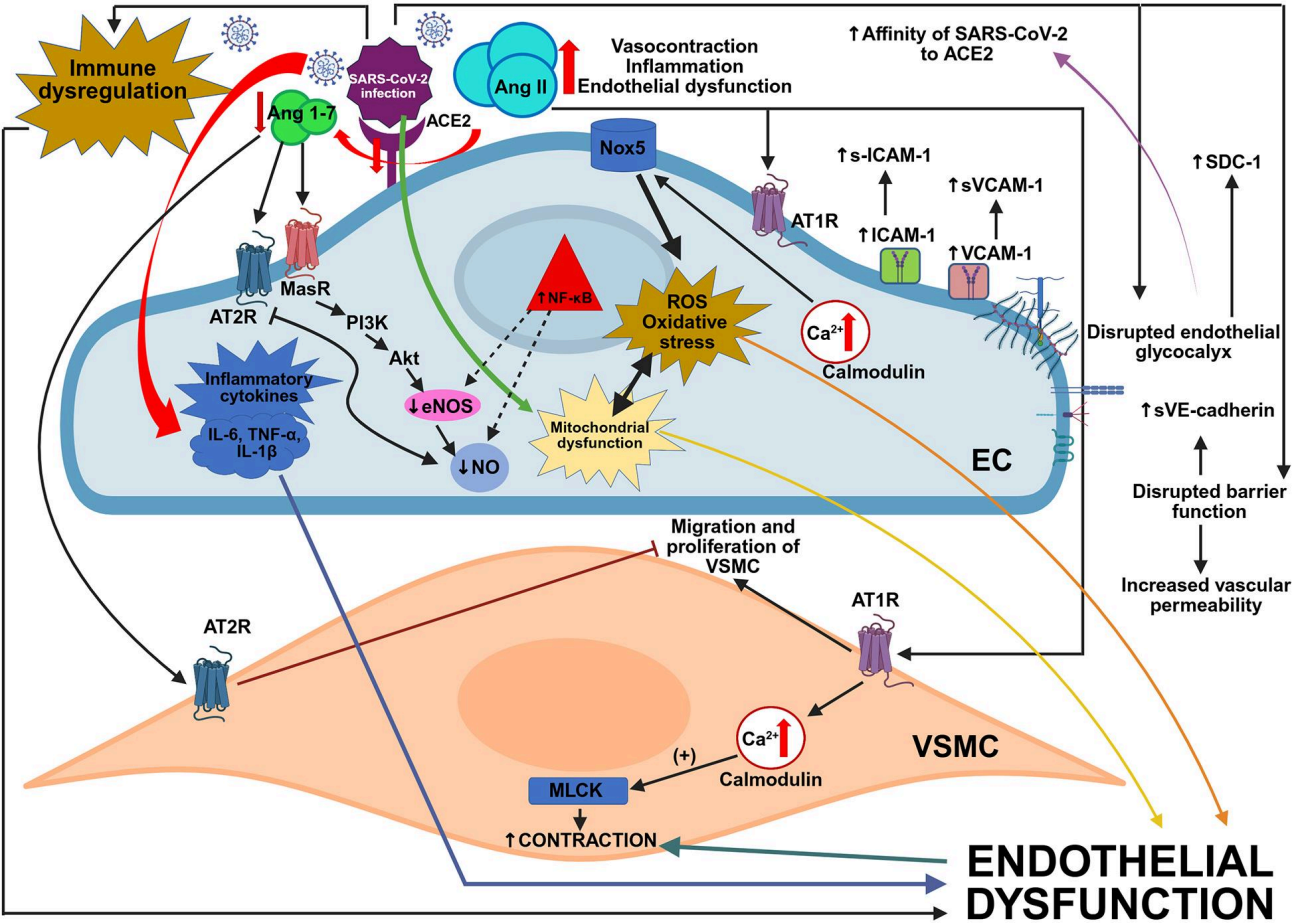
SARS-CoV-2 infection and endothelial dysfunction. ACE2, angiotensin-converting enzyme 2; Ang II, angiotensin II; Ang 1-7, angiotensin 1-7; AT1R - angiotensin type 1 receptor; AT2R - angiotensin type 2 receptor; EC, endothelial cell; eNOS, endothelial nitric oxide synthase; ICAM-1, intercellular adhesion molecule 1; IL-6, interleukin 6; IL-1β, interleukin 1β; MasR, Mas receptor; MLCK - myosin light-chain kinase; NF-κB, nuclear factor kappa B; NO, nitric oxide; NOX5, nicotinamide adenine dinucleotide phosphate (NADPH) oxidase 5; ROS- reactive oxygen species; SDC-1, syndecan-1; sICAM-1, soluble intercellular adhesion molecule 1; sVCAM-1, soluble vascular cell adhesion molecule 1; sVE-cadherin, soluble vascular endothelial cadherin; TNF-α, tumor necrosis factor alpha; VCAM-1, vascular cell adhesion molecule 1; VSMC, vascular smooth muscle cell.

In summary, the pathomechanisms of acute COVID-19 infection in the CV system include direct viral toxicity, intracellular viral entry via ACE2 and glycocalyx modulation, alteration of ACE2 signaling cascade towards the activation of harmful Ang II/AT1R axis, activation of pro-inflammatory proteins and deregulation of the immune system, hyper-inflammatory milieu and “cytokine storm” formation, hyper-coagulopathy development, ED and damage (Figure 2), and cardiac fibrosis and cardiac remodeling (23, 114). Mechanisms responsible for the subsequent development of LC include virus-specific pathomechanisms described above, aberration of immune reaction and development of OS and inflammation-mediated cellular damage (Figure 2) as a reaction to acute infection, and expected sequelae of post-critical illness (67).

## 5 Prevention and treatment

Since the pandemic started, preventive tools that could help prevent infection or a severe course of the disease have been discussed daily by the specialists and the lay public. Now that the pandemic situation has been tamed, due to vaccination, prevention and/or treatment begins to be perceived from a different angle, precisely from LC's point of view. Here we will discuss preventive tools that can be used considering the CV system. Preventing COVID-19 is an important strategy for patients with CV risk factors (20).

As mentioned above, LC has a multifactorial pathology, in which the literature mentions some causes, such as SARS-CoV-2 persistence in body tissue reservoirs; immune dysregulation with or without reactivation of the underlying pathogens, microbiota dysbiosis, redox abnormalities, autoimmunity, microvascular blood coagulation, endothelial and mitochondrial dysfunction as well as dysfunctional signaling in the brainstem and/or vagus nerve, this combined with risk factors such as type 2 DM, sex (mainly female), ethnicity (of Latino origin), socioeconomic factors (low income), exposure to COVID-19 reinfection, the presence of specific antibodies, connective tissue disorders, and attention deficit hyperactivity disorder, however, one-third of people with LC have no preexisting conditions identified (115). Public health practices, including handwashing, social distancing, proper ventilation, air filtration for public spaces, and mask-wearing, are a cornerstone in reducing the spread of viral infections and minimizing their impact on health. Combined with vaccination, they have mitigated the effects of the COVID-19 pandemic (116). The impact of vaccination on the incidence of LC differs across studies. Some studies showed, that vaccination against COVID-19 decreases the chance of LC symptoms (5, 117). Similarly, the impact of vaccination on LC symptoms in LC patients differs among cases, with 16.7% of patients experiencing relief of symptoms, 21.4% experiencing a worsening of symptoms, and 61.9% experiencing unchanged symptoms (118). Vaccination against COVID-19 may prevent CVDs in some cases, however, rarely may cause myocarditis or pericarditis, especially in young males, though at a lower rate than following SARS-CoV-2 infection (20). In addition, patients with post-COVID-19-mRNA vaccination myocarditis, contrary to patients with post-COVID-19 myocarditis, have a lower frequency of CV complications than those with conventional myocarditis at 18 months (119). An analysis of the US Department of Veterans Affairs national healthcare databases suggests that vaccination before infection confers only partial CV protection six months after the acute phase of the disease (120). A large observational study showed that patients who had been vaccinated and then became infected with SARS-CoV-2 had a lower incidence and burden of LC than those who were unvaccinated and became infected (121). On the other hand, there is evidence that reinfections increase the risk of LC, including CV disorders even in vaccinated people (11). It is well-known that vaccination against COVID-19 represented a clear reduction in both mortality and ICU admissions (122) and a recent large study of the English adult population has found that the incidence of CV events, such as strokes, heart attacks, and blood clots, was lower after COVID-19 vaccination (123). Vaccination has also been shown to reduce the risk of cardiac injury and was associated with lower post-COVID cardiac complications and symptoms (122). However, the impact of old vaccination and boosters on the incidence of CV complications in the context of new SARS-CoV-2 variants of concern is unclear. Several studies also indicate that vaccination before SARS-CoV-2 infection reduces the risk and severity of LC symptoms, particularly fatigue and neurocognitive complaints (124–126). However, Al-Aly et al. observed that vaccination does not fully protect against CV complications following COVID-19 (120). This discrepancy may reflect the multifactorial origin of cardiovascular damage, including endothelial and microvascular injury, persistent immune dysregulation, and RAAS imbalance that may not be adequately addressed by vaccine-induced systemic immunity. Therefore, vaccination should be regarded as a key preventive tool that mitigates systemic inflammation and overall disease burden but may require complementary endothelial-targeted or metabolic interventions to prevent cardiovascular sequelae.

Because LC affects many millions of people and because the cases still rising and have become increasingly worrying, an urgent need has arisen for effective treatments against this disease. However, the number and pace of clinical trials to tackle LC are vastly insufficient (127). Therefore, it is essential to identify molecules and drugs capable of fighting against LC symptoms. There is a thorough list of potential treatments, including antivirals, immunomodulators, anticoagulants or platelet inhibitors, and modulators of metabolism, CV and neurological function, or other organ-specific functions. However, most need to be clinically trialed. Currently, LC does not have effective validated treatments (115). Although there are currently no broadly effective specific treatments for LC, many are under investigation, and certain components have been effective for subsets of LC populations (5). The proposed treatment strategies involve pharmacological and non-pharmacological approaches, including β-blockers, ivabradine, pyridostigmine, fludrocortisone, midodrine, increased salt and fluid intake, intravenously administered salt, compression stockings for treatment of POTS, Coenzyme Q_10_, D-ribose for fatigue, anticoagulants and apheresis for abnormal clotting, Sulodexide and Pycnogenol for ED (5, 128). Given that LC may be related to an abnormally regulated immune system various immunotherapy, including immunosuppressors and immunomodulators have been suggested for LC symptoms treatment (13, 16).

The antiviral drug nirmatrelvir-ritonavir (Paxlovid) during acute infection decreased the incidence of LC (16). Patients with POTS and IST may benefit from low-dose beta-blocker therapy for heart rate management and reducing adrenergic activity (67).

SARS-CoV-2 infections are also associated with higher profibrotic dipeptidyl peptidase-4 (DPP-4)-mediated mechanisms and suppression of AMP-activated protein kinase (AMPK - adenosine monophosphate-activated protein kinase) activation in kidney cells. Lowered DPP-4 levels and restoration of AMPK levels are organ-protective, suggesting a pathogenic role of DPP-4 and a protective role of AMPK in diabetic COVID-19 patients (129). Renoprotective strategies targeting the ACE2/Ang 1-7 axis and the modulation of AMPK activity have shown promise in experimental models for mitigating renal damage associated with COVID-19 (129). AMPK is also a potential target for therapeutic intervention and preventive strategies in other cardiometabolic diseases (130). AMPK activation can mitigate ED by reducing OS and inflammation and improving vascular health (130). Additionally, in the randomized-controlled trial, the diabetes drug metformin (activator of AMPK) initiated within 7 days of SARS-CoV-2 infection reduced the risk of LC by 41% (131). In a retrospective cohort analysis prevalent metformin use was associated with a lower incidence of LC in adults with type 2 DM (132). However, further studies are needed to analyze the use of AMPK activators in diabetic and nondiabetic LC patients.

In addition to pharmacological strategies, spa rehabilitation for patients with LC is also recommended (29). It was shown, that a high-altitude mountain environment with spa rehabilitation significantly improved clinical symptoms, lung functions, and platelet mitochondrial bioenergetics in patients with LC (29). Physical exercise is one of the most frequently used by specialists, including WHO-recommended preventive tools for decreasing the risk of several chronic diseases such as CVDs. It was shown that physical exercise improves insulin and leptin sensitivity, reestablishes BP values, decreases blood viscosity, and increases NO production (133). Cardiovascular protection from exercise is attributed to AMPK activation. AMPK is a crucial cellular energy sensor, phosphorylating several metabolic signaling pathways to ensure cell survival (134, 135). AMPK is also an enzyme phosphorylating eNOS, so its activation increases NO production (136) and improves endothelial function (137). The cardioprotective role of α1AMPK, a predominantly expressed vascular isoform, is emphasized under OS conditions and vascular inflammation (138–140). Thus, activation of α1AMPK could represent a preventive and/or therapeutic strategy against the CV complications seen in LC. On the other hand, exercise, cognitive behavioral therapy, and graded exercise therapy are contraindicated in LC patients with post-exertional malaise (5) which is associated with a worsening of fatigue- and pain-related symptoms after acute mental or physical exertion (141). Appelman, Charlton (141) showed that skeletal muscle structure is associated with a lower exercise capacity in LC patients, and local and systemic metabolic disturbances, severe exercise-induced myopathy, and tissue infiltration of amyloid-containing deposits in skeletal muscles of patients with LC worsen after induction of post-exertional malaise (141).

### 5.1 Treatment related to RAAS

Clinical efforts focus on repurposing already approved drugs for the treatment of LC symptoms. Although current pharmacological therapies against acute and post-acute COVID-19 mainly centered on blocking viral replication and limiting inflammation, the novel therapeutic approaches targeting ED could represent a promising strategy for CV sequelae in LC patients (142). Several categories of endothelium-targeted therapies, such as lipid-lowering (statins), hypoglycemic (metformin, SGLT2 inhibitors), anti-coagulatory (heparin), anti-hypertensive (ACEI/ARBs), antioxidant (vitamin C, flavonoids), anti-inflammatory (glucocorticoids, tocilizumab, anakinra, colchicine, JAK inhibitors), and other drugs (ET-1 receptor blockers, ACE2 agonists, L-Arginine, NO donors, fluvoxamine, traditional Chinese medicine, senolytics) have potential to improve ED in COVID-19 patients (23).

The pathogenesis of SARS-CoV-2 infection involves a dysregulation of the RAAS (143). Therapeutics that target the RAAS appear to be promising to treat LC symptoms. RAAS can be modulated via several inhibitors and activators. In the context of LC pharmacological agents inhibiting harmful Ang II/AT_1_R axis by angiotensin receptor blockers (ARB), angiotensin-converting enzyme inhibitors (ACEI), and or pharmaceuticals which are activating protective ACE2/Ang 1-7/AT_2_R axis, for example – Compound 21 (C21), can be used. ARB and ACEI are commonly used for hypertension and heart failure treatment (144). Initially was believed, that ACEIs and ARBs could increase the vulnerability to SARS-CoV-2 by upregulating the expression of ACE2 (23). ACE2 is often upregulated when comorbidities are present (145). Also, ACE inhibition can play a role during infection, inhibition decreases Ang II production with the subsequent reduction of its vasoconstrictor effect, inflammation, and fibrosis inhibition (146, 147). ARB administration is not only a blocker of AT_1_R but also can increase the expression of ACE2 and thus increase the production of protective Ang 1-7 (148). On the other hand, ACE2/Ang 1-7/AT_2_R stimulation seems to have a more considerable therapeutical potential due to the RAAS imbalance during the COVID-19 described above. A meta-analysis analyzed the impact of ACEI/ARB treatment on the clinical outcomes of patients with COVID-19 among the population in the East-Asia region by Huang, Yuan (149). They showed that ARB and ACEI use reduced the hospitalization duration and mortality (149). Until now, there is no evidence that RAAS blockers: ACEIs, or ARBs increase CV complications or worsen prognosis (13). On the other hand, ACEI and ARBs to treat hypertension are less effective in the black population and they have been shown to have adverse effects as well. Moreover, COVID-19 is more severe in the racial minorities (145).

Treatment by C21, an AT_2_R ligand, reduces fibrosis, hypertrophy of the right heart ventricle, and expression of pro-inflammatory cytokines leading to cardiac function improvement, moreover it can improve COVID-19-induced lung injury, lung dysfunction, and associated cardiopulmonary pathology (150). Activators of ACE2 – xanthenone and Mas receptor agonist – CGEN-856S are promising agents for hypertension treatment and ameliorating myocardial fibrosis. They induced vascular repair and improved endothelial function, but this effect was observed only in animal models. Their effectiveness in the treatment of COVID-19 and LC has not been proven yet (151–153). An exciting group of RAAS modulators are biased AT_1_R ligands, such as β-arrestins selectively binding on AT_1_R. The treatment with β-arrestins reduces BP and improves cardiac function (154). Due to the possibility of their use in the treatment of LC, this question, also because LC is a new and not fully understood topic, is not yet answered, and further investigation is necessary. However, due to their use in the treatment of CVDs, their use can represent a suitable therapeutic tool.

### 5.2 Nutrition and dietary recommendations

It was proved that patients with LC are, from a nutrition point of view characterized by malnutrition and consecutive loss of fat-free mass (155). Therefore, for the management of LC, it is more than necessary to reestablish daily calorie intake but also to balance the composition of the diet considering their nutritional value and the inclusion of foods that help in the treatment and prevention of LC. It is known that increased fruit and vegetable consumption is linked with several beneficial effects, including cardioprotection. Increased intake of fruits and vegetables can reduce the risk of developing CV disease (156, 157). A healthy and balanced diet is, among other things, a source of vitamins, minerals, and phytochemicals, which not only have cardioprotective effects but also can reduce OS and inflammation, playing a role in LC development. Micronutrient supplements such as vitamin C, vitamin D, and zinc are often used in managing viral illnesses (158). Although contradictory data exist, supplementation with several vitamins and minerals is suggested, for reducing the risk of infection for their “immune-supportive” properties. Their deficit could contribute to an impaired immune reaction and, last but not least, to longer-lasting LC symptoms (159). It must be mentioned that the substances described below are not intended to treat LC. Still, their use can help to prevent LC development or help to treat it in combination with traditional pharmacological medication. Moreover, many nutrients and natural substances in exceedingly high doses can be dangerous (160). Still, the clinical significance of micronutrients in patients with LC remains unclear. Yet, only a few ongoing registered clinical trials are evaluating the effect of different micronutrient supplements on the clinical outcomes of LC patients.

#### 5.2.1 Vitamin C

Vitamin C, an essential micronutrient with antioxidant properties and cofactor for biosynthesis and gene regulatory enzymes, affects redox homeostasis and the immune system. It regulates neutrophil chemotaxis, phagocytosis, B and T cell differentiation and proliferation, and microbial killing. It is necessary for neutrophil apoptosis, thereby reducing necrosis and potential tissue damage (161, 162). During acute infection, plasma levels of vitamin C are reduced due to increased metabolic demands (160, 162). It was shown extremely low vitamin C levels in critically ill patients with COVID-19 (163). Therapeutic effects during acute infections have been a matter of debate, with conflicting results in studies of respiratory infections and critically ill patients (164). A meta-analysis published by Milani, Macchi (164) studied the supplementation of vitamin C as a prevention strategy for COVID-19 patients. They have shown that despite the partial results indicating an improvement in health status after supplementation with vitamin C, no complete and clear conclusion can be drawn due to the incompleteness of some studies, and some are still waiting for the results (164). Another study by Xia, Qin (165) in Wuhan showed that high-dose intravenous vitamin C reduces pro-inflammatory cytokine levels (IL-6, IL-8, TNFα) and mitigates myocardial damage as a result of hyperinflammation triggered by COVID-19 infection (165). However, vitamin C supplementation had no significant effect on mortality, intubation rate, and length of hospital stay of COVID-19 patients in the meta-analysis of Beran, Mhanna (158). A meta-analysis showing the effect of vitamin C supplementation on LC is not yet available (116). However, there is a single-blind randomized, placebo-controlled study on the combination therapy of vitamin C and L-arginine in adults with persistent fatigue after COVID-19. This co-therapy improved endothelial function, fatigue, muscle strength, and walking performance (166). Vitamin C could also affect the binding of SARS-CoV-2 with ACE2. The deubiquitinating enzyme USP50 regulates the ubiquitination and stability of the ACE2 protein. Vitamin C blocks the USP50-ACE2 interaction thereby promoting ACE2 degradation and restricting SARS-CoV-2 entry (167). Vitamin C supplementation could also help prevent/treat LC concerning CV comorbidities. The CV's protective actions of vitamin C due to its antioxidant properties are very well described (168). In detail, vitamin C-induced inhibition of LDL oxidation can reduce atherosclerosis (169). Besides that, vitamin C reduces monocyte adhesion in smokers (170), resulting in endothelium protection. Endothelial function is modulated via vitamin C and its role in NO production (171) and ROS reduction (172). In the study of d'Uscio et al. (171), the beneficial effect of vitamin C on vascular endothelial function appears to be mediated in part by the protection of BH4 and restoration of eNOS enzymatic activity.

The BH4 deficiency and OS are also hypothesized in LC patients (84). In addition, hemorheological properties represent significant contributors to the pathogenesis of CVDs. Physical changes in blood cells, including reduced erythrocyte deformability are considered to play a role in COVID-19-related vascular occlusion and organ damage. The persisting changes in blood cell physical phenotypes could contribute to the long-term impairment of circulation and oxygen delivery linked with COVID-19 and could be connected with long-term symptoms of the recovered patients (173). Radosinska, Jasenovec (174) observed promotion in whole blood rheology because of improved erythrocyte deformability in healthy young volunteers taking vitamin C (1,000 mg per day) for 3 weeks (174). As improvement in hemorheology may play a key role in cardioprotection, it would be challenging to investigate vitamin C supplementation for patients suffering from LC symptoms in randomized controlled trials. Still, no conclusion can be drawn on the efficacy of vitamin C in preventing COVID-19 and LC (116).

#### 5.2.2 Vitamin D

Vitamin D regulates calcium and phosphate metabolisms and maintains a normal mineralized skeleton. Besides that, it was shown that the active form of vitamin D – 1,25-dihydroxy vitamin D participates in the immune system due to modulation of the innate and adaptive immune system and suppression of inflammatory processes. Vitamin D deficiency is connected with several immune-related diseases and disorder developments, including psoriasis, type 1 DM, sepsis, respiratory infections, etc. (175). Clinical studies showed that vitamin D deficiency is associated with a higher risk of respiratory infections, including COVID-19 (116). Moreover, vitamin D can modulate RAAS via renin expression inhibition and activation of the ACE2 signaling pathway (176). Like vitamin C, the level of vitamin D and its metabolites are decreased in COVID-19 patients, and this reduction correlates with the severity level of acute infection (160). It was shown low plasma levels of vitamin D in critically ill patients with COVID-19 (163). Studies also showed that low serum levels of vitamin D and zinc are associated with poor outcomes in patients with COVID-19 (158) and a good vitamin D status may have beneficial effects on the course of the disease (177, 178). Among hospitalized patients with moderate to severe COVID-19, severe vitamin D deficiency was associated with a trend for longer hospital length of stay (179). It was proved that supplementation helps with SARS-CoV-2 negativization (180), connects with decreased mortality of infected COVID-19 patients (177, 181), and is associated with a lower risk of hospitalization (182). Therefore, it could be expected that supplementation may be an effective tool in the prevention of LC development and/ or treatment. However, in the meta-analysis of Beran, Mhanna (158) vitamin D supplementation was not associated with a mortality benefit in COVID-19, albeit showed a lower intubation rate and shorter hospital stay length than standard-of-care. This meta-analysis demonstrated consistent results on mortality in subgroup analysis of studies supplemented with vitamin D pre- or post-COVID-19 diagnosis (158).

Even if it turns out that vitamin D supplementation can inhibit hyperinflammation and accelerate the recovery process (183), evidence about the preventive role of vitamin D against LC is missing. Vitamin D has its place in the CV system, and its deficiency is linked to CV complication development (184). There is a link between vitamin D deficiency and adverse vascular remodeling, ED, vascular inflammation, and increased risk for CV and cerebrovascular diseases (184). Vitamin D affects BP due to its negative regulation of the harmful RAAS axis. Still, a direct relationship has not been confirmed yet, and it is proposed that vitamin D deficiency is linked to hypertension development (185). It was reported that vitamin D regulates the expression of genes involved in cellular proliferation and differentiation and thus modulates angiogenesis and vascular remodeling (186). Moreover, it is suggested that the vitamin D receptor is necessary for endothelial function homeostasis (187). In vitamin D deficiency the endothelium-mediated vasorelaxation was impaired due to reduced eNOS expression and NO production (184). Vitamin D has also been reported to upregulate the expression of eNOS, increase the dimer-to-monomer ratio of the eNOS protein, and modulate the phosphorylation of eNOS, leading to increased eNOS activity and, thus, enhanced NO production (184). Vitamin D can also modulate the AMPK signaling pathway resulting in eNOS phosphorylation in endothelium (188). Yet, vitamin D administration seemed to not influence the prevention of LC symptoms at month six among people aged 16 years and older (189). Townsend, Dyer (190) investigated the relationship between vitamin D concentrations and the cardinal features of LC, fatigue, and reduced exercise tolerance in 149 patients at a median of 79 days after COVID-19. The results suggest that persistent fatigue and reduced exercise tolerance following COVID-19 are independent of vitamin D levels (190). Pizzini, Aichner (191) found no association between vitamin D deficiency and long-term COVID-19 outcomes (191). Indeed, vitamin D seems to provide some benefits in the long-term complications of COVID-19 (192). A randomized controlled trial (151 adult patients, duration of 24 weeks) showed that the combination of vitamin D₃ (2,000 IU/day) and K₂ reduced the number of LC symptoms, inflammatory markers, and OS, while being safe and well tolerated (193). This information, therefore, points to the possibility that vitamin D supplementation, especially in the case of its deficiency, which has been observed because of COVID-19, could be an effective way to help fight against LC, specifically CV comorbidities that are related to LC.

#### 5.2.3 Zinc

Zinc is one of the most important micronutrients for life. Zinc is a trace element that has been shown to play a significant role in both innate and adaptive immune systems and cytokine production (158). It participates in important cellular processes such as cellular differentiation, proliferation, transport, DNA synthesis, reproduction, and regulation of the endocrine, immune, and central nervous systems (CNS). It interacts with more than 300 enzymes and 2,000 transcriptional factors (194). Zinc affects the function of the immune system due to the modulation of the skin barrier and gene regulation within lymphocytes. Zinc is crucial for immune cells' normal development and function, responsible for non-specific immunity, such as neutrophils and natural killer cells (195). Moreover, zinc is an essential component of numerous enzymes, such as antioxidant superoxide dismutase (196). Zinc deficiency is related to increased susceptibility to various pathogens, resulting in dysfunctional humoral and cell-mediated immunity. Moreover, zinc can modulate DNA replication, RNA transcription, apoptosis, cell division, and activation and has antioxidant properties (195). On the other hand, zinc inhibits virus replication, prevents fusion with the host cell membrane, inhibits the activity of virus polymerases, and interferes with viral protein synthesis (160). It has been shown that a combination of zinc and a zinc-ionophore pyrithione could inhibit the replication of the SARS-CoV virus *in vitro* via virus polymerase inhibition (197). Zinc supplementation in COVID-19 patients was reported to reduce mortality, as shown in a meta-analysis published by (198). However, in another meta-analysis, zinc supplementation was not associated with a significant reduction in mortality among COVID-19 patients (158). The role of zinc in the treatment and prevention of LC should also be considered since there is evidence that zinc concentrations are decreased and correlate with the severity and progression of chronic fatigue syndrome triggered by viral infection (199). Zn status is an important feature of CV health (200). Imbalance in Zn homeostasis is also linked to CV disease development, such as coronary heart disease, ischemic cardiomyopathy, myocardial infarction, etc. (194, 201). Also, Zn deficiency leads to the progression of type 2 DM (194). A recent double-blind, placebo-controlled randomized trial showed beneficial effects of the consumption of 2 cps/day, for two months, of an oral food supplement (OFS), based on Echinacea angustifolia, rosehip, propolis, royal jelly, and zinc, in LC patients. Results showed the OFS's beneficial effects on the inflammatory state, fatigue, and quality of life in LC patients (202). The results of these reports suggest that some patients with LC should be treated with zinc supplements. However, further studies are needed to show that zinc can treat LC symptoms and LC-related CV diseases.

#### 5.2.4 Selenium

Selenium is an essential trace element important for redox homeostasis regulation due to selenocysteine, which is present in the catalytic center of several selenoproteins (203). Selenium deficiency affects inflammation and immune response due to the modulation of immune cell activation, differentiation, and proliferation. This deficiency, combined with OS, can affect how the host organism perceives the pathogen, resulting in a normally mildly pathogenic virus becoming a highly virulent agent. Also, higher pathogenicity and mortality under selenium deficiency were observed for SARS-CoV-2 (204). Interestingly it was observed the interaction between the main virus COVID-19 protease (responsible for virus replication) and antioxidant enzyme – glutathione peroxidase-1, the enzyme-containing selenium in the catalytic center, and this interaction depends on selenium concentration, indicating the importance of selenium in the SARS-CoV-2 infection process (205). On the other hand, because selenium is an essential factor for the normal functioning of several antioxidant enzymes (glutathione peroxidase, thioredoxin reductase, etc.), its deficiency can affect homeostasis forward to OS, which can lead to cellular/tissue damage and hyper-inflammation as well. Fakhrolmobasheri, Mazaheri-Tehrani (206) have shown that selenium deficiency was not only present in COVID-19 patients but also connected with the more severe course of acute infection. Those results indicate that selenium supplementation could help to prevent disease progression (206), especially in critically ill patients. Moreover, significant clinical benefits of selenium supplementation have been demonstrated in other viral infections (204). Yet, the need to keep normal selenium concentration is even more emphasized during hyper-inflammation and long-term OS, which is probably responsible for LC development. Since selenium deficiency can disturb normal CV homeostasis, lack of selenium contributes to the pathogenesis of multiple CVDs, including myocardial infarction, heart failure, coronary heart disease, and atherosclerosis (207). Interestingly, selenium deficiency was first associated with Keshan's Disease, characterized by cardiomyopathy and heart failure (207). Selenium, as part of selenoproteins, is crucial for the CV system due to its involvement in ROS elimination and detoxification, resulting in cardiac and endothelial function maintenance (208). Here evidence about the effect of selenium on LC is missing. Further investigation should be especially focused on the CV system and LC-related comorbidities.

#### 5.2.5 Vitamin A

Vitamin A is also essential for the innate and adaptive immune cell response and the general immune system (209). It is necessary for maintaining healthy respiratory and intestinal epithelial barriers (210). Vitamin A is a so-called “anti-infection vitamin” due to its role in the resistance of the host to infection, which is reduced by vitamin A deficiency. Deficiency affects the amount and activity of cells of the innate and adaptive immune system (lymphocytes, macrophages, granulocytes, natural killers). It was shown that vitamin A metabolite – all-trans retinoic acid, can increase the expression of ACE2. This could have a double effect, especially at the time of SARS-CoV-2 exposure (210). First, it could increase the risk of infection because the ACE2 receptor is reported as a critical factor for intracellular entry of the virus (211). Second, the supplementation of vitamin A could prevent sympathetic nervous system activation during the severe course of infection via protective ACE2 pathway activation (210, 212). Also, its metabolites regulate immune response, such as retinol affecting immune response via its autocrine and paracrine regulation. In the context of COVID-19 as a respiratory infection, vitamin A is an essential micronutrient for normal lung development and repair (210). Therefore, vitamin A deficiency during COVID-19 infection can increase the risk of severe inflammation type 1 development, cause severe lung-tissue damage, and prevent damaged tissue repair. Tepasse, Vollenberg (209) showed that vitamin A levels were significantly lower in hospitalized COVID-19 patients than in convalescent persons. Of the hospitalized patients, those who were critically ill showed significantly lower vitamin A levels than those who were moderately ill. Besides that, vitamin A deficiency correlated with lymphopenia, a marker of COVID-19 acute infection severity level (209). However, further studies are needed to clarify the role of vitamin A during COVID-19, as no conclusion could be drawn on its efficacy in COVID-19 management (116). In one Czech study, Krcmova, Javorska (213) showed that the serum concentrations of vitamins A, D, and E were not different between hospitalized survivors and patients who died from COVID-19. The authors also detected low vitamin A, E, and D levels during hospitalization as compared to levels with control healthy volunteers (213). However, there were no differences in the vitamin levels between patients with or without LC symptoms. Concentrations of β-carotene (provitamin A) in blood were lower in patients with LC symptoms (29). Additionally, the level of retinoic acid correlates with CV risk. In detail, low levels were linked to increased mortality in patients with coronary artery disease. Moreover, it is proposed that vitamin A is essential for heart regeneration, postnatal cardiac function, and CV disease progression (214). This suggests a potential aspect of vitamin A supplementation in LC treatment and CV homeostasis regulation. Further studies are needed to clarify the role of vitamin A during COVID-19 and LC, as only a few studies are available on vitamin A as a potential LC preventive/therapeutic tool.

#### 5.2.6 Coenzyme Q10

Coenzyme Q10 (CoQ10), an endogenously produced and vitamin-like compound, is responsible for electron transport between mitochondrial complexes, and its presence is necessary for aerobic respiration and mitochondrial adenosine triphosphate (ATP) production (215). It also acts as a cofactor in several processes, including biosynthesis, catabolism, detoxification, cell signaling, and others (216). Mitochondrial function, dynamics, and metabolism could be affected by COVID-19 infection. That was confirmed by an experiment where the mitochondrial function was determined in mononuclear cells isolated from the peripheral blood of COVID-19-positive patients. Mitochondrial dysfunction, altered mitochondrial morphology and bioenergetics, and altered cellular metabolism with increased glycolysis were found (217–219). SARS-CoV-2 manipulation of mitochondrial function may persist for an extended time after acute COVID-19 (220, 221). Also, Sumbalova, Kucharska (221) showed that the SARS-CoV-2 virus can modulate platelet mitochondrial respiration, oxidative phosphorylation, and reduced endogenous CoQ10 production in patients after acute COVID-19 (221). It was proposed that mitochondrial dysfunction could result in CoQ10 deficiency due to OS-modulated endogenous biosynthesis. Mitochondrial dysfunction triggered by OS because of COVID-19 infection can be prevented by CoQ10 supplementation. Similarly, it was proposed that CoQ10 supplementation can be applied in prolonged OS resulting in LC development (29, 222, 223). Kucharska, Sumbalova (29) showed accelerated recovery of patients with LC syndrome after mountain spa rehabilitation and a reduced form of CoQ10, ubiquinol supplementation 2 × 100 mg daily. In this study, increased systemic and cellular CoQ10 concentration alleviated clinical, including CV symptoms, and improved antioxidant protection of the patients. Moreover, the working group of Prof. Gvozdjakova draws attention to the importance of monitoring and ensuring adequate levels of CoQ10 in LC (29). However, Hansen, Mogensen (218) observed only a slight trend toward CoQ10 having potentially beneficial effects in LC patients (218). Coenzyme Q10 is also used for the supplementary treatment of CVDs (heart failure, hypertension, ED, etc.) (224, 225). In addition, the deficit of CoQ10 was observed in patients with coronary artery disease (226). Supplementation by CoQ10 has vast potential in the field of CVD treatment such as atherosclerosis and hypertension, and its use has a positive effect on vascular stiffness and ED (224, 225). Therefore, the use of CoQ10 for LC prevention and/or treatment should be considered regarding its effect on the CV system.

#### 5.2.7 Tetrahydrobiopterin (BH4)

It was hypothesized, that upon exposure to SARS-CoV-2, the innate immune system may quickly induce inducible nitric oxide synthase (iNOS), thereby causing the production of excessive superoxide because iNOS is uncoupled by the marginal deficiency of BH4 in the patients with mutations of GCH1 (GTP cyclohydrolase 1) and related genes. The resulting OS becomes self-perpetuating, even when the virus has been cleared because the deficiency of BH4 that initially triggered the OS now also directly impairs the immune regulators responsible for deactivating that ongoing OS. A hypothesized BH4 deficiency in LC patients with exogenous BH4 to recouple iNOS, eNOS, and nNOS and restore oxidative balance can be translated into clinical treatment (84).

#### 5.2.8 Flavonoids

Among natural antioxidants, the most frequently examined compounds are the flavonoids, once known as vitamin P. Flavonoids are a group of natural polyphenolic substances. Various experimental and epidemiological studies have shown that the consumption of flavonoid-rich foods is associated with a reduced risk of CVDs (227). They can be found in various foods, particularly in olive oil, fruits, including citruses, vegetables, green and black tea, and cocoa, as well as in red wine and beer and were extensively tested for their potential cardioprotective effects in cardiometabolic diseases (227, 228). The natural polyphenols have broad anti-inflammatory, endothelial protective, and anti-thrombotic effects, and other mechanisms argue for a potential beneficial effect of these substances on LC pathological mechanisms. Despite growing evidence that proved the role of natural polyphenols in supporting the immune system, the debate about the use of natural agents in the prevention and management of LC is still far from solved. Some studies suggested a clinical benefit of some flavonoids in managing COVID-19 due to their antimicrobial and immunomodulatory properties.

A green tea-derived epigallocatechin-3-gallate (EGCG) has a wide spectrum of antiviral activity, also against SARS-CoV-2 (229). Frank, Dickinson (230) showed that epigallocatechin-3-gallate-palmitate nanoformulations possess strong antiviral activity against human coronaviruses and could be developed as an intranasally delivered new drug to eliminate the persistent SARS-CoV-2 infection, leading to restored olfactory function and reduced inflammation in the CNS (230). Nanoformulations containing epigallocatechin-3-gallate-palmitate showed properties compatible with nasal application to rapidly inactivate SARS-CoV-2 residing in the olfactory mucosa and to reduce inflammation in the CNS (231, 232). EGCG is a strong antioxidant and has anti-inflammatory activity, and it has also been shown to provide cardioprotective effects (231).

(–)-Epicatechin is a flavonoid with antihypertensive effects and multiple beneficial actions in the CV system, but it also possesses antiviral effects (227). Epicatechin has shown promising effects in the treatment of CVDs (233). Several studies, including ours, suggest that epicatechin improves endothelial function (227, 233). Epicatechin possesses antioxidant, anti-fibrotic, anti-thrombotic, anti-aggregatory, anti-diabetic, and anti-inflammatory properties, and also inhibits RAAS and elevates NO production (227). Epicatechin, which is structurally like estrogen, seems to be a major bioactive cocoa flavanol. Cacao beans are the most abundant source of epicatechin. Radosinska, Horvathova (234) found, that acute ingestion of dark chocolate improved erythrocyte deformability (234). Increased deformability of erythrocytes may improve the rheological properties of blood and thus hemodynamics in humans, resulting in better tissue oxygenation. Previously it has also been shown that epicatechin exhibited endothelium-dependent and independent vasodilatory effect (227). Epicatechin is a well-tolerated, relatively non-toxic, and bioavailable flavonoid (227). Moreover, treatment in the form of nanoparticles provides a promising way to increase the bioavailability of epicatechin (227). In our studies, epicatechin has an antihypertensive effect, and improved erythrocyte deformability and endothelial function (233, 235). Thus, epicatechin could be relevant to the treatment of CV complications in LC patients.

Quercetin is another well-known flavonoid whose antiviral properties have been investigated in numerous studies. Interestingly, quercetin and vitamin C co-administration exerts a synergistic antiviral action. Studies suggest that its supplementation may promote antioxidant, anti-inflammatory, and immunoprotective effects. It has been suggested to be a key mediator in the CV protective element of the Mediterranean diet. Quercetin inhibits SARS-CoV 3CL protease by binding to its Gln189 site, which is expressed similarly by SARS-CoV-2 and therefore provides a direct mechanistic rationale for its clinical use in acute COVID-19 (162), and also in LC patients (115) in addition to its immunoprotective, anti-inflammatory, and antioxidant actions. Moreover, quercetin exerted cardioprotective effects, in both preclinical animal and clinical studies (228). It has been shown that SARS-CoV-2 stimulates mast cells. Natural flavonoids, such as quercetin, could be used as mast cell inhibitors, they inhibit neuroinflammation and decrease cognitive decline (160). The study of Jasenovec, Radosinska (236) indicates the potential benefit of quercetin treatment on erythrocyte deformability and NO production in the condition of DM (236). Indeed, further studies are necessary to confirm quercetin's beneficial activities in LC patients. Numerous studies have shown that natural flavonoids modify multiple cellular and molecular mechanisms that may be significant in terms of the prevention and treatment of LC. However, to date, there remains a lack of clinical studies.

## 6 Conclusions

SARS-CoV-2 can result in several CVDs, both during acute infection and following recovery as a late complication. There is no doubt about the need for a deeper understanding of the pathophysiology of LC. Biomedical research identifies various pathophysiological changes; however, many questions remain unanswered. This review discusses the pathomechanisms of CV damage in COVID-19, especially in LC patients, and provides some suggestions for preventive and therapeutic strategies. Because LC can impair patients' ability to work, attend school, take care of family, and care for themselves, resulting in profound emotional and physical effects on the patients, their families, and caregivers (4) guide research toward the identification of pathomechanisms for preventive and therapeutic purposes is desirable. Information provided in this review article regarding the CV damage of LC patients is based on current evidence and draws attention that more research is needed about the pathophysiological features of this complex condition. The long-term pathophysiology across organ systems after newer SARS-CoV-2 variants remains to be elucidated. SARS-CoV-2 infection is still having a devastating impact on many aspects of human life, on the healthcare system, economy, social relations, and mental health, among others. Although the emergency phase is over, the pandemic is not over. The sequelae caused by the SARS-CoV-2 virus also continue. Many questions remain and are a priority to address, therefore urgent research, which requires an integrated approach rather than organ or disease-specific, is needed to establish LC and help LC patients.

Cost-effective, globally available, safe, and effective therapeutic interventions are warranted to ameliorate the morbidity and mortality of LC patients. Currently, there is no specific drug or other method to minimize LC-associated CV symptoms. Thus, there is an urgent need to develop an effective approach to treat CV symptoms and diseases associated with LC. Among the potential therapeutic approaches, interventions targeting endothelial dysfunction (such as RAAS modulators, and flavonoids) and persistent inflammation (e.g., antioxidants, vitamin C, vitamin D, and AMPK activators like metformin) appear particularly promising. In patients with coagulation-related phenotypes, anticoagulant or antiplatelet strategies may offer benefits, whereas those with dysautonomia or POTS may respond better to β-blockers or volume-expanding agents. Future research should focus on tailoring treatment based on the predominant underlying mechanism, integrating endothelial, immunologic, and metabolic phenotyping to achieve precision management of long COVID-related cardiovascular sequelae.

Agents that target immune modulation and have anti-viral and endothelium-protective effects may present exciting targets for pharmacological intervention of LC patients. The use of micronutrients and natural substances is a relatively inexpensive and easily manageable treatment. If proven effective, it has the potential to change the LC course and provide great hope for countering the LC symptoms. However, further research is needed to better understand the clinical significance of these supplements in LC patients.
